# Factors Responsible for Prehospital Delay in Patients with Acute Coronary Syndrome in Bangladesh

**DOI:** 10.3390/medicina58091206

**Published:** 2022-09-02

**Authors:** Md. Fakhrul Islam Khaled, Dipal Krishna Adhikary, Md. Mazharul Islam, Md. Mashiul Alam, Mohammad Walidur Rahman, MSI Tipu Chowdhury, Roseyat Perveen, Sharmin Ahmed, Eshita Ashab, Shiblee Sadeque Shakil, Sanjida Ansari, Bikash Chandra Das, Noor Mohammad, Mohammad Abul Ehsan, Abu Baqar Md. Jamil, Zahidul Mostafa, Zainal Abedin, Sajal Krishna Banerjee

**Affiliations:** 1 Department of Cardiology, Bangabandhu Sheikh Mujib Medical University, Dhaka 1000, Bangladesh; 2 Ministry of Municipality, Doha P.O. Box 35081, Qatar; 3 Internal Medicine, Bridgeport Hospital, Yale University, 30 Coleman st Apt#11, Bridgeport, CT 06604, USA; 4 National Institute of Cardiovascular Diseases, Dhaka 1207, Bangladesh; 5 Chattogram General Hospital, Chattogram 4000, Bangladesh; 6 Sheikh Hasina Medical College, Tangail 1900, Bangladesh; 7 Anwar Khan Modern Medical College, Dhaka 1209, Bangladesh; 8 Shaheed Syed Nazrul Islam Medical College Hospital, Kishorganj 2300, Bangladesh; 9 Mymensingh Medical College Hospital, Mymensingh 2200, Bangladesh; 10 Nepal Medical College Teaching Hospital, Kathmandu 13344, Nepal; 11 Dhaka Medical College, Dhaka 1312, Bangladesh; 12 M Abdur Rahim Medical College, Dinajpur 5200, Bangladesh; 13 Sylhet MAG Osmani Medical College, Sylhet 3100, Bangladesh; 14 Shaheed Ziaur Rahman Medical College, Bogra 5800, Bangladesh; 15 Cox’s Bazar Medical College, Cox’s Bazar 4700, Bangladesh; 16 Lalmonirhat District Hospital, Lalmonirhat 5500, Bangladesh; 17 University Grants Commission, Dhaka 1207, Bangladesh

**Keywords:** acute coronary syndrome, prehospital delay, Bangladesh

## Abstract

*Background:* Acute coronary syndrome (ACS) remains a cause of high morbidity and mortality among adults, despite advances in treatment. Treatment modality and outcomes of ACS mainly depend on the time yielded since the onset of symptoms. Prehospital delay is the time between the onset of myocardial ischemia/infarction symptoms and arrival at the hospital, where either pharmacological or interventional revascularization is available. This delay remains unacceptably long in many countries worldwide, including Bangladesh. The current study investigates several sociodemographic characteristics as well as clinical, social, and treatment-seeking behaviors, with an aim to uncover the factors responsible for the decision time to get medical help and home-to-hospital delay. *Materials and Methods:* A prospective cross-sectional study was conducted between July 2019 and June 2020 in 21 district hospitals and 6 medical college hospitals where cardiac care facilities were available. The population selected for this study was patients with ACS who visited the studied hospitals during the study period. Following confirmation of ACS, a semi-structured data sheet was used to collect the patient data and was subsequently analyzed. *Results:* This study evaluated 678 ACS patients from 30 districts. The majority of the patients were male (81.9%), married (98.2%), rural residents (79.2), middle-aged (40–60 years of age) (55.8%), low-income holders (89.4%), and overweight (56.9%). It was found that 37.5% of the patients received their first medical care after 12 h of first symptom presentation. The study found that the patients’ age, residence, education, and employment status were significant factors associated with prehospital delay. The patients with previous myocardial infarction (MI) and chest pain arrived significantly earlier at the hospital following ACS onset. Location of symptom onset, first medical contact with a private physician, distance from symptom onset location to location of first medical contact, the decision about hospitalization, ignorance of symptoms, and mode of transportation were significantly associated with prehospital delay. *Conclusions*: Several factors of prehospital delay of the ACS patients in Bangladesh have been described in this study. The findings of this study may help the national health management system identify the factors related to treatment delay in ACS and thus reduce ACS-related morbidity and mortality.

## 1. Introduction

Coronary artery disease (CAD) accounts for 32% of all global deaths, representing the leading cause of adult mortality [1]. Acute coronary syndrome (ACS) refers to a group of symptoms comprising unstable angina and myocardial infarction (MI) with or without ST-segment elevation [2]. It is the leading cause of ischemic heart disease (IHD)-related deaths, representing 1.8 million deaths per year, and remains a source of high morbidity and mortality despite advances in treatment [3]. Treatment modality and success of ACS mainly depend on the time yielded since the onset of symptoms. Delays between symptom onset and treatment of less than 60 min are desirable [4]. Mortality and morbidity are directly related to the time between symptom onset and initiation of treatment [5]. Prehospital delay is the time between the onset of myocardial ischemic symptoms and arrival at the hospital, where pharmacological or interventional revascularization is available. This delay remains unacceptably long in many countries worldwide, including Bangladesh [6,7,8].

A greater understanding of the contributing factors for prehospital delay may aid in reducing these delays. The total prehospital delay period includes the time taken by patients to recognize the seriousness of their symptoms and to contact medical help (decision time) and the time taken from requesting help to admission to a center where emergency coronary care service is available (home-to-hospital delay) [4]. Different factors may affect these two components. In-hospital delay, also known as door-to-treatment, is the time from hospital arrival to initiation of reperfusion therapy [4,5]. Regardless of shortening in-hospital delay, reperfusion therapy cannot achieve the best results if the prehospital delay is not reduced. Most studies evaluating prehospital delay were conducted in developed countries. Several sociodemographic and clinical factors, including older age, female sex, low socioeconomic status, previous cardiac illness history, and comorbidities, were related to longer prehospital delay [5]. Other factors associated with greater delay included self-medication or consultation with non-qualified people, insufficient knowledge about ACS, and underestimation of the seriousness of an MI.

Bangladesh is currently a middle-income country with a state of progressive economic development. Parallel to the rising economy, the life expectancy of the country’s people is increasing over time [9,10]. Disease patterns are shifting from communicable to non-communicable diseases (NCD). A survey in 2020 demonstrated that 9 of the top 10 causes of death were due to NCDs. IHD is one of the leading causes (25.9%) of adult male mortality in Bangladesh [11]. Great diversity exists among the sociodemographic, educational, and cultural characteristics of Bangladeshi populations. Due to an absence of a uniform system for its delivery, access to primary health care is highly variable amongst different population groups. The country is divided into 64 administrative districts, each of which has several upazilas. The upazila governmental hospitals (health complexes and health centers) are equipped with primary health care facilities to run preventive health care services and to serve certain routine and emergency diseases. District hospitals provide primary and secondary facilities. Medical college hospitals and several specialized government hospitals are situated in different districts and divisions, and the capital city of Dhaka serves tertiary medical facilities. In addition, there are many private hospitals situated in different parts of the country, mostly with secondary and often tertiary health care facilities. The majority of tertiary level and super-specialized private hospitals are situated in big cities like Dhaka, Sylhet, Khulna, and Chattogram. People initially seek treatment from either nearby government or private physicians, but many rely on non-qualified health practitioners or drug sellers for initial care. A national health insurance system does not currently exist in Bangladesh. However, physician consultations, certain basic investigations, and limited medications are available in a government hospital for free or at minimum cost. Additionally, a centralized referral system is absent in this country. A patient himself decides his point of consultation, either an unqualified health care provider or a tertiary-level hospital. A patient’s socioeconomic status is also an essential factor in their access to and selection amongst different levels of hospital care. Treatment facilities for ACS are not uniformly available across the country. Primary percutaneous coronary intervention (PCI) is primarily available in larger cities. Thrombolysis is available in medical colleges and in some district hospitals. However, such services are not available at many district hospitals. For these reasons, the prehospital delay for patients with acute MI may be considerably longer.

A number of studies were conducted in Bangladesh to identify the factors responsible for the prehospital delay of ACS patients. However, all were single-center studies in different parts of the country, such as the north-east, north-west, south-east, and central regions [12,13,14,15,16,17]. The current research investigated sociodemographic, clinical, social, and treatment-seeking behavior in different medical colleges and secondary- and tertiary-level hospitals across the country. We set out to discover what factors are associated with the three components of decision time, home-to-hospital delay, and first medical contact to revascularization delay. We hypothesized that prehospital delay time is significantly associated with sociodemographic characteristics of patients, previous clinical history, distance, and the healthcare-seeking behavior of the patients.

## 2. Materials and Methods

### 2.1. The Study Settings

**Figure 1:**
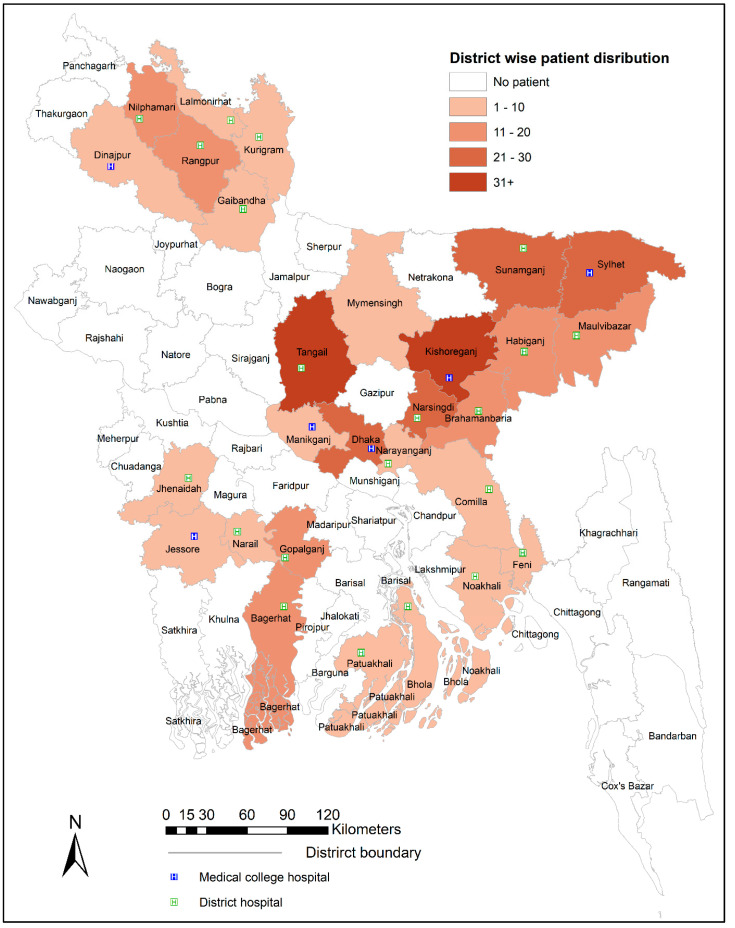
The map of Bangladesh depicting the studied locations.

A prospective cross-sectional study was conducted between July 2019 and June 2020 in 21 district hospitals with tertiary facilities and 6 medical college hospitals where cardiac care facilities (CCU) were available (Figure 1). The minimal inclusion criteria for the CCU were the presence of a cardiologist, cardiac wards, and the capability to diagnose and manage ACS, including reperfusion with injection of streptokinase.

### 2.2. Patient Selection

Every patient with ACS who visited the studied hospitals during the study period was initially selected in this study. ACS included ST-elevated MI, non-ST-elevated MI, and unstable angina. MI was defined as a rise and/or fall of elevated cardiac troponin-I value with at least one of the following two criteria: (i) patients having symptoms suggestive of myocardial ischemia and (ii) ECG changes consistent with MI [18]. The respective consultants confirmed the diagnosis of ACS. Other inclusion criteria included the ability to recall the time of symptom onset, duration of time between symptom onset and calling for medical help and events before hospital admission. Patients having comorbid conditions (renal failure, cancer, stroke, ongoing infection, or inflammatory conditions such as inflammatory bowel disease) that might influence symptom presentation or troponin positivity were excluded from this study.

### 2.3. Data Collection

Two stakeholders from each hospital were selected for patient selection and data collection. An orientation meeting was conducted with the stakeholders to explain the objectives of the study, the data collection procedure, the patient’s rights, and the ethical considerations of the research. Each patient was explained the study objectives, possible outcomes, and benefits of the research and was asked to participate in the research voluntarily. If a patient was interested in volunteering for the research, he/she was informed about their rights and responsibilities in sharing information. Subsequently, a semi-structured data sheet was prepared to collect the patient data. Each dataset had four sections: patient consent form, patient demographic and socioeconomic details, clinical histories, and clinical profile. Medical records of a patient were examined in his presence for validation of the records and to collect clinical data for the research. 

### 2.4. Statistical Analysis

The patient data were entered in a Microsoft Excel 2016 spreadsheet, and statistical analyses were performed using RStudio version 4.1.2. Descriptive statistics were performed to determine the percentage (%) and 95% confidence interval (CI) of every variable. Univariate and multivariate analyses were performed to assess the association between different sociodemographic characteristics, chronic diseases, and other risk factors with prehospital delay (<12 h/earlier and >12 h/delayed for hospitalization) by chi-squared (χ^2^) and logistic regression tests, respectively. The *p*-value (<0.05) was considered a significant variation among the variables.

## 3. Results

### 3.1. Demographic Information of the Patients

This study evaluated 678 patients with ACS from 30 districts (Figure 1) of Bangladesh. The patient demographic profiles are presented in Table 1. The majority of the patients were male (81.9%, 95% CI: 78.78–84.58), married (98.2%, 95% CI: 96.93–98.98), rural residents (79.2, 95% CI: 75.98–82.09), middle-aged (40–60 years) (55.8%, 95% CI: 51.99–59.45), low-income holders (89.4%, 95% CI: 86.84–91.48), and overweight (56.9%, 95% CI: 53.18–60.61). It was found that 37.5% (258/678, 95% CI: 33.89–41.17) of the patients received their first medical care after 12 h of first symptom onset.

### 3.2. Association between Sociodemographic Characteristics and Prehospital Delay

A total of five variables were analyzed to identify the sociodemographic causes of prehospital delay in ACS patients (Table 2). The study found that age, location of residence, education level, and employment status of the patients were significant factors for prehospital delay. Delay to hospitalization increased with the increase in age of the patients, although it was not significant by logistic regression. Approximately 29% of the patients below 40 years of age were transferred to the hospital after 12 h of the first onset of symptoms, whereas for the aged patients (>60 years), this value was 53.7%. The percentage of patients hospitalized after 12 h of ACS onset was 41% and 23% in rural vs. urban areas, respectively. The study revealed that the patients’ education level was directly proportional to the rate of early hospitalization. The percentage of hospitalization within 12 h of the onset of symptoms in illiterate, primary, secondary, or higher level educated patients were 57.1%, 66%, 59.2%, and 86.4%, respectively. Unemployment was associated with increased prehospital delay, with 67.7% of patients hospitalized after 12 h of ACS onset. On the other hand, 82% of the job holders were hospitalized within 12 h.

### 3.3. Association between Chronic Diseases and Prehospital Delay

We studied twelve chronic disease conditions with possible associations with prehospital delay (Table 3). Two of these conditions—prior myocardial infarction and the presence of chest pain—were found to be significantly associated with prehospitalization delay. About 48.5% of the patients with a history of myocardial infection arrived late at the hospital. It was found that among the patients with chest pain, 62.7% were admission to the hospital earlier (<12 h).

### 3.4. Association between other Miscellaneous Factors and Prehospital Delay

Some miscellaneous factors were also found to be associated with prehospital delay (Table 4). Patients who developed ACS at home (vs. workplace) were more likely to have delayed hospitalization (62.4%). About 60% of patients whose FMC was registered by private physicians experienced delayed time to admission. Residing closer to an FMC was associated with lower rates of delayed admission. Of the patients who delayed making the decision on whether to get medical help, 67.6% were hospitalized late. Of those who ignored cardiac symptoms, 66.2% experienced delayed care. Patients with previous hospitalization records were less likely to experience a delayed admission vs. those with no such history (62.2% vs. 37.6%). The use of an ambulance was found to be a significant mode of transportation associated with reduced delay to hospitalization, whereas public transportation was associated with increased prehospital delay.

## 4. Discussion

Identifying possible risk factors of a disease is necessary for guiding its diagnosis, therapy, and control [8,19]. The present study identified several demographic, socioeconomic, cultural, clinical, and health service system factors that impacted the time lag from the onset of symptoms to arrival at a CCU experienced by ACS patients in Bangladesh. Most of the study population was above 40 years of age. Previous studies found that increased age is associated with a longer prehospital delay [8,20,21], which is in accordance with the current study. Several factors may contribute to such delay, including (i) the elderly’s reduced physical activity and a lower ability to perceive pain; (ii) increased likelihood of atypical symptom presentation and increased prevalence of comorbidities in older patients, which may result in a delay in seeking medical care [6,22] and decreased ability to recognize warning symptoms [23] and (iii) inadequate perception of risks associated with an acute MI and the increased wish to avoid burdening family members seen in elderly populations [24]. The study suggests that ACS is a male-dominant condition. Female patients were admitted to the hospital earlier than the males (although the association was not significant), a finding which may signify an evolving change in social gender norms and an improvement in gender discrimination in Bangladesh. Some studies reported that female patients with AMI had a more prolonged decision-making process than males [8,25]. In contrast, other studies have not found significant gender differences [26,27]. 

A significant finding in the current study was the increased incidence of delayed hospitalizations in patients from rural areas. This raises particular alarm regarding inadequate access to medical care, as most health care services in Bangladesh are urban based, whereas most of its population lives in rural areas. Rural people receive primary treatment from unqualified health care providers, resulting in specific management delays. In addition, education can be associated with this type of delay. Rural residents in Bangladesh typically achieve lower levels of education than urban residents [28]. This finding is supported by other studies, which demonstrated that low education levels, poor employment status, and poor economic conditions are significant predictors of longer prehospital delay [29]. In addition, patients with health insurance have a shorter wait than those without insurance [30]. Similar to our findings, a previous study reported that patients with prior AMI experienced decreased delay to hospitalization [31], but others did not find any association between delay and history of AMI [32]. Patients with ACS presented with various symptoms, such as chest pain, breathlessness, and palpitation. Patients with chest pain tended to present to the hospital earlier. This finding correlates with the common perception that ischemic heart disease presents with chest pain. Different studies observed that patients with atypical symptoms had substantially longer prehospital delays [33,34]. Patients with diabetes, hypertension, renal impairment, thyroid dysfunction, smoking, or angina pectoris had longer prehospital delays than those without these conditions. A partial explanation may be that elderly patients with multiple comorbidities often have altered perceptions of pain and may present with atypical symptoms, resulting in a denial of the ischemic symptoms that may result in this delay. 

An observation noted in this study was that the place where symptoms were perceived first significantly affected the prehospital delay. Being at home or in a public place when symptoms began resulted in a longer delay in treatment [35]. On the other hand, people tend to remain alert about health risks in the workplace. However, one study found no significant differences in prehospital delay time depending on where the symptoms began [36]. Patients with symptoms alone experienced longer delays than those who experienced them in the company of others [37]. Having someone present when the symptoms occur can reduce fear and lead to responsible decision-making regarding treatment and, thus, a shorter prehospital delay [21]. Health service-seeking behavior and the health service system greatly impact prehospital delay. The current study signifies the comparative strength of the Bangladesh governmental upazila health complexes over private health services. The upazila health complexes are more organized than private services at this practitioner level concerning appropriate referral. Patients who presented to upazila or district hospitals were more likely to get earlier diagnosis and treatment than those who presented to private physicians. Self-treatment with drugs or rest can lead to a long prehospital delay, which was also observed in a previous study [30]^.^ On the other hand, patients who had previously been hospitalized were notified and presented earlier [38,39]. Similar to other studies [8,39,40], this study also revealed that those who used ambulances were more likely to reach the cardiac center early on than those who used public transport and self-transport. Appropriate public health measures can be taken with the findings of the current study to reduce prehospital delay in ACS, which is essential to improve the outcome of management.

## 5. Conclusions

This study reveals that several sociodemographic factors in Bangladesh, such as rural residence, lower level of education, and unemployment status of a patient, are significantly associated with prehospital delay. Place of the first appearance of symptoms, nature of symptoms, first medical contact, distance from symptoms to first medical contact, a delayed decision about hospitalization, lack of symptom understanding, and mode of transportation significantly impact prehospital time. Actions should be taken to reduce the prehospital delay of ACS patients in Bangladesh. Health education and awareness development may be beneficial. However, further interventional research to reduce prehospital delay is warranted. The results of this study can be used for further research, development, and policymaking in the national health management system to aid in identifying the factors associated with treatment delay in ACS and thus reduce ACS-related morbidity and mortality.

## Figures and Tables

**Table 1:** Demographic characteristics of the study patients (N = 678).

Characteristics		*n* (%, 95% Confidence Interval)
Gender	Female	123 (18.1, 15.42–21.22)
	Male	555 (81.9, 78.78–84.58)
Marital Status	Single	3 (0.4, 0.15–1.29)
	Married	666 (98.2, 96.93–98.98)
	Widow	9 (1.3, 0.69–2.50)
Residence	Rural	537 (79.2, 75.98–82.09)
	Urban	129 (19.0, 16.25–22.15)
Education	Illiterate	315 (46.5, 42.74–50.22)
	Primary	150 (22.1, 19.16–25.40)
	Up to SSC	147 (21.7, 18.74–24.93)
	Above SSC	66 (9.7, 7.72–12.20)
Occupation	Unemployment	93 (13.7, 11.33–16.51)
	Day labor	204 (30.1, 20.76–33.65)
	housewife	105 (15.5, 12.96–18.40)
	Job	66 (9.7, 7.72–12.20)
	Business	204 (30.1, 26.76–33.65)
Age	<40 years	84 (12.4, 10.11–15.08)
	40–60 years	378 (55.8, 51.99–59.45)
	>60 years	216 (31.9, 28.46–35.46)
Income (Bangladeshi Taka)	<20,000	606 (89.4, 86.84–91.48)
	20,000–30,000	51 (7.5, 5.77–9.76)
	>30,000	21 (3.1, 2.03–4.69)
Body Mass Index	Normal	292 (43.1, 39.39–46.82)
	Overweight	356 (56.9, 53.18–60.61)
Prehospital delay	<12 h	420 (61.9, 58.23–65.52)
	>12 h	258 (37.5%, 33.89–41.17)

**Table 2:** Association between sociodemographic characteristics and prehospital delay.

Variables	Categories	N	Prehospital Delay		*p*-Value	
			<12 h (*n*, %)	>12 h (*n*, %)	Univariate Analysis	Multivariate Analysis
Gender	Female	123	84 (68.3)	39 (31.7)	0.135	0.242
	Male	555	339 (61.1)	216 (38.9)		
Age	<40	84	60 (71.4)	24 (28.5)	0.001	0.127
	40–60	378	200 (52.9)	178 (47.1)		
	>60	216	100 (46.3)	116 (53.7)		
Residence	Rural	537	318 (59.2)	219 (40.8)	0.001	0.001
	Urban	129	99 (76.7)	30 (23.3)		
Education	Illiterate	315	180 (57.1)	135 (42.9)	0.001	0.001
	Primary	150	99 (66)	51 (34)		
	Secondary	147	87 (59.2)	60 (40.8)		
	Above Secondary	66	57 (86.4)	9 (13.6)		
Employment	Unemployment	93	30 (32.3)	63 (67.7)	0.001	0.001
	Day labor	204	120 (58.8)	84 (41.2)		
	Housewife	105	72 (68.6)	33 (31.4)		
	Job	66	54 (81.8)	12 (18.2)		
	Business	204	150 (73.5)	54 (26.5)		

**Table 3:** Association between chronic diseases and prehospital delay with acute coronary syndrome.

Variables	Categories	N	Prehospital Time		*p*-Value	
			<12 h (*n*, %)	>12 h (*n*, %)	Univeriate Analysis	Multivariate Analysis
History of Mycordial Infarction	Absent	579	375 (64.8)	204 (35.2)	0.011	0.024
	Present	99	51 (51.5)	48 (48.5)		
Hypertesion	Normotensive	381	240 (63)	141 (37)	0.714	0.272
	Hypertensive	297	183 (61.6)	114 (38.4)		
Diabetes Mellitus	Non-diabetic	468	285 (60.9)	183 (39.1)	0.231	0.152
	Diabetic	210	138 (65.7)	72 (34.3)		
Dyslipidaemia	Absent	531	327 (61.6)	204 (38.4)	0.409	0.228
	Present	147	96 (65.3)	51 (34.7)		
Family History	No history	489	294 (60.1)	195 (39.9)	0.051	0.049
	History	189	129 (68.3)	60 (31.7)		
Smocking	Non-smoker	243	153 (62.9)	90 (37.1)	0.850	0.339
	Smoaker	405	252 (62.2)	153 (37.8)		
Chronic Kidney Disease	No	642	399 (62.1)	243 (37.9)	0.586	0.399
	Yes	36	24 (66.7)	12 (33.3)		
Thyroid Disorder	Hyperthyroid	45	24 (53.3)	21 (46.7)	0.409	0.271
	Euthyroid	615	387 (62.9)	228 (37.1)		
	Hypothyroid	18	12 (66.7)	6 (33.3)		
Physical Activity	Sedentary	198	114 (57.6)	84 (42.4)	0.156	0.110
	Regular activity	306	192 (62.7)	114 (37.3)		
	Irregular activity	174	117 (67.2)	57 (32.8)		
Chest Pain	Absent	273	105 (38.5)	168 (61.5)	0.001	0.001
	Present	402	252 (62.7)	150 (37.3)		
Breathlessness	Absent	513	321 (62.6)	192 (37.4)	0.618	0.281
	Present	159	96 (60.4)	63 (39.6)		
Palpitation	Absent	612	384 (62.7)	228 (37.3)	0.560	0.274
	Present	66	39 (59.1)	27 (40.9)		

**Table 4:** Association between different factors and prehospital delay with acute coronary syndrome.

**Variables**	**Categories**	**N**	**Prehospital Time**		***p*-Value**	
			**<12 h** **(*n*, %)**	**>12 h** **(*n*, %)**	**Univariate Analysis**	**Multivariate Analysis**
Place of first onset of symptom	At home	558	210 (37.6)	348 (62.4)	0.001	0.001
	At work	120	75 (62.5)	45 (37.5)		
Status of first medical contact	Upazila	153	108 (70.6)	45 (29.4)	0.001	0.074
	District	75	54 (72)	21 (28)		
	Medical college	315	207 (65.7)	108 (34.3)		
	Private physician	135	54 (40.0)	81 (60.0)		
Distance from place of symptom to first medical contact	<5 km	366	240 (65.6)	126 (34.4)	0.001	0.001
	<15 km	126	48 (38.1)	78 (61.9)		
	>15 km	186	60 (32.3)	126 (67.7)		
Delay in making decision to get medical help	No delay	363	192 (52.9)	171 (47.1)	0.001	0.001
	Delay	102	33 (32.4)	69 (67.6)		
Ignorance of symptoms	Not ignored	288	162 (56.3)	126 (43.8)	0.001	0.001
	Ignored	177	60 (33.8)	117 (66.2)		
Previous hospitalization	No history	543	204 (37.6)	339 (62.4)	0.001	0.001
	History	135	84 (62.2)	51 (37.8)		
Mode of transportation	Ambulance	39	36 (92.3)	3 (7.7)	0.012	0.001
	Private	516	333 (64.5)	183 (35.5)		
	Public	36	15 (41.6)	21 (58.4)		

## Data Availability

All data are available from the first author.
